# Perforated Jejunal Diverticulitis: Surgical and Antibiotic Management

**DOI:** 10.7759/cureus.66952

**Published:** 2024-08-15

**Authors:** Erin G Park, Kylie Besly, Anna Kim, Colton P Boney, Sharon Mbuko

**Affiliations:** 1 Medicine, Alabama College of Osteopathic Medicine, Dothan, USA; 2 Biomedical Sciences, Geisinger Commonwealth School of Medicine, Scranton, USA; 3 Internal Medicine, Crestwood Medical Center, Huntsville, USA

**Keywords:** surgery and infectious disease, antibiotic treatment for diverticulitis, small bowel diverticulitis, small bowel diverticulosis, jejunal diverticulitis

## Abstract

Small bowel diverticulitis occurs at a rate of 0.06% to 1.3%, mainly in individuals over 50, peaking between ages 60 and 70. Duodenal diverticula are the most common (79% of cases), followed by jejunal or ileal diverticula (18%), and diverticula in all segments combined (3%). This condition typically presents with sporadic abdominal pain and vague gastrointestinal symptoms, making diagnosis difficult. We report an 80-year-old male who presented to the emergency department with sudden, left-sided abdominal pain and nausea due to perforated jejunal diverticulitis. Despite undergoing side-to-side jejunojejunostomy and incidental appendectomy, the patient rapidly declined and expired 45 hours post-operation due to septic shock. This case highlights the scarcity of literature on jejunal diverticulitis and its treatment guidelines.

## Introduction

Jejunal diverticulitis is a rare condition, occurring in 0.3%-2.3% of cases of abdominal pain, characterized by the herniation of mucosa and submucosa through weak points in the small intestine, typically along the mesenteric border [1]. Its pathogenesis remains unclear, although hypotheses suggest abnormalities in smooth muscle function, myenteric plexus integrity, intestinal dyskinesia, and elevated intraluminal pressures [2].

Among these small intestinal diverticula, duodenal diverticula are more frequent, followed by diverticula of the jejunum and ileum. Small intestinal diverticula predominantly affects individuals over 50 years of age, with a peak occurrence between 60 to 70 years [3]. Autopsy studies have shown incidences between 1.3% and 4.6% while radiologic studies show an incidence between 0.02% and 2.3% [4]. Most cases are located in the proximal jejunum, followed by the distal jejunum [2]. Jejunal diverticulitis often presents with intermittent abdominal pain and nonspecific gastrointestinal symptoms, making diagnosis challenging. Laboratory findings typically show leukocytosis and elevated inflammatory markers, mimicking other gastrointestinal conditions like acute appendicitis, colonic diverticulitis, or Crohn’s disease.

Our case study involves an 80-year-old male presenting to the emergency department with nausea and sudden-onset left abdominal pain. He required empirical antibiotic treatment and emergent side-to-side jejunostomy. However, postoperatively, the patient developed a fever, and despite multiple attempts at cardioversion and resuscitation, he passed away 45 hours after surgery. This study focuses on the management of symptoms and clinical findings observed during the patient’s hospitalization. Strategies such as infectious disease consultations in cases of sepsis have been shown to reduce in-hospital mortality rates [5]. Given the rarity of symptomatic jejunal diverticulitis, treatment guidelines are limited, posing challenges for physicians.

## Case presentation

An 80-year-old obese male arrived at the emergency department complaining of nausea and abdominal discomfort. He complained of a gradual onset of left-sided abdominal pain while at rest, with no accompanying symptoms reported. The patient reported a pain level of 8 out of 10. His vitals were considered stable with a blood pressure of 129/79, heart rate of 90 bpm, respiratory rate of 18, temperature of 98.6 F, and oxygen saturation (SpO2) at 95% at the emergency department. At this time, the patient did not meet systemic inflammatory response syndrome (SIRS) criteria (0 points). The patient denied using tobacco or any illicit drug usage. The patient noted drinking one to two beers every night. 

**Figure 1:**
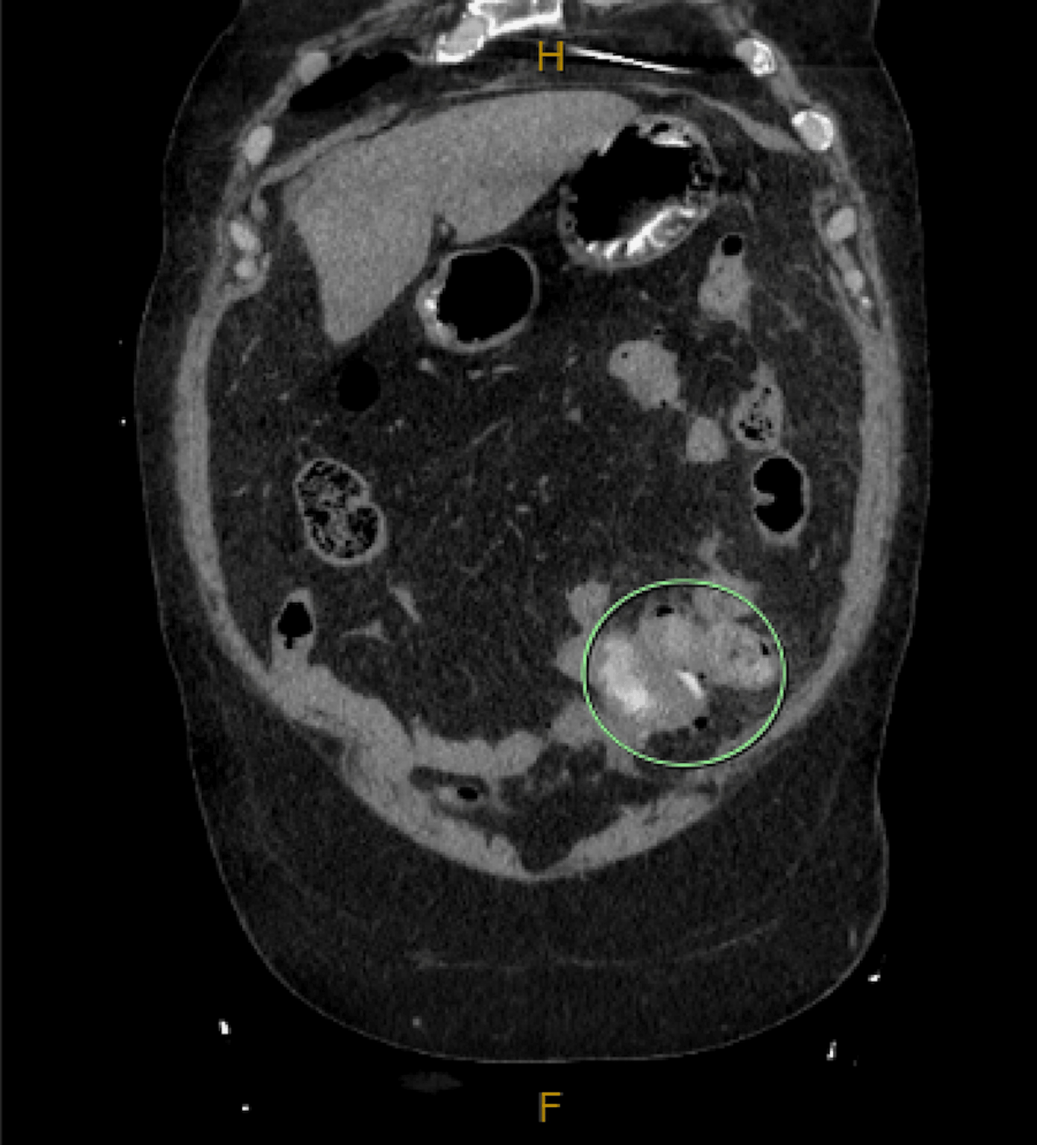
Coronal computed tomography (CT) image of the abdomen with oral and intravenous contrast showing mesenteric inflammation and intraperitoneal free air consistent with perforated small bowel diverticulitis

**Figure 2:**
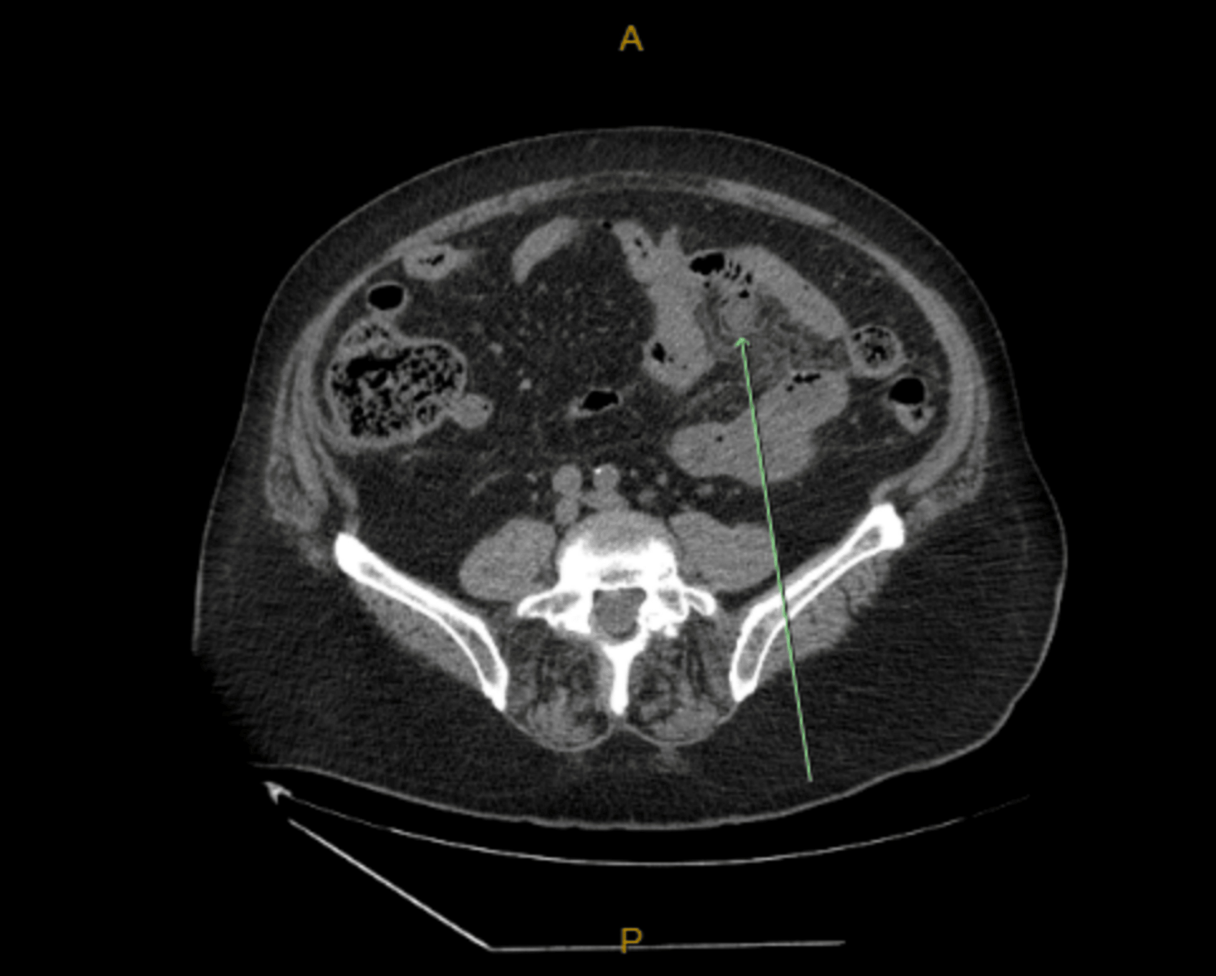
Axial computed tomography (CT) image of the abdomen with oral and intravenous contrast showing mesenteric inflammation and intraperitoneal free air consistent with perforated small bowel diverticulitis

**Figure 3:**
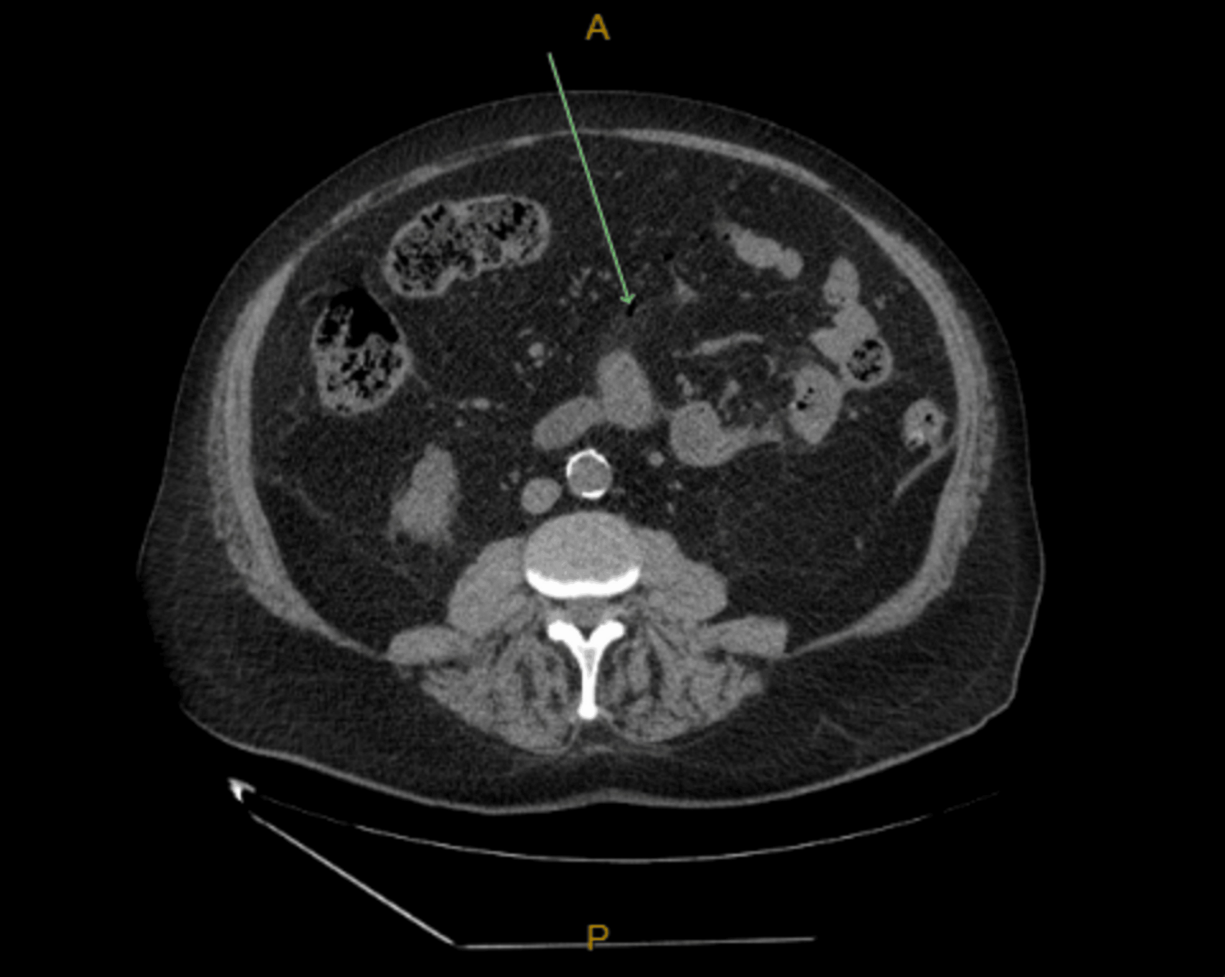
Axial computed tomography (CT) image of the abdomen with oral and intravenous contrast showing sigmoid diverticulosis

His past medical history includes acute cholangitis, asthma, chronic kidney disease (CKD) stage 3, congestive heart failure, coronary arteriosclerosis, gastroesophageal reflux disease (GERD), hyperlipidemia, and hypertension. After reviewing medical records, the patient was diagnosed with diverticulosis in 2018 after a routine colonoscopy. His surgical history consisted of endoscopic retrograde cholangiopancreatography, implantation of an internal cardiac defibrillator, coronary artery stent placement, transurethral prostatectomy, and cholecystectomy. Laboratory studies showed increased levels of blood urea nitrogen (BUN)/creatinine (Cr) (baseline Cr of 1.8 mg/dL), leukocytosis of 19,100 cells/μL with a left shift (predominant neutrophils), hemoglobin at 15.3 g/dL (normal 12-16), and a platelet count of 150,000 (130,000-400,000). Differential diagnoses consisted of gastritis, diverticulitis, and colitis. A computed tomography (CT) scan of the abdomen confirmed the diagnosis of perforated small bowel diverticulitis and sigmoid diverticulosis (Figures 1-3).

The patient was empirically treated with piperacillin/tazobactam (Zosyn), and general surgery was consulted. Intraoperatively, the surgeon found a perforated proximal jejunal diverticulum and an interloop abscess in the left upper quadrant. The surgeon then performed a side-to-side jejunostomy and the mesenteric defect at the jejunostomy was closed. The abdominal cavity was irrigated with several more liters of normal saline mixed with vancomycin. Postoperatively, the patient began to show signs of septic shock with a temperature of 101.1 °F, heart rate of 163 bpm, respiratory rate of 22, blood pressure of 99/64, and SpO2 of 94%. Meropenem was added during this time. The patient ultimately expired after 13 cardioverter-defibrillator firings and cardiopulmonary resuscitation.

Pathology of the appendix confirmed focal acute appendicitis with marked acute serositis. The portions of the small bowel showed both acute and chronic diverticulitis with reactive changes and serositis.

## Discussion

Diverticular disease, especially that of the colon, is a common cause of hospitalization in the United States. Small bowel manifestations are rare, with those occurring in the jejunum being rarer still; of the total number of presentations for small bowel diverticular disease, only 18% was present in the jejunum or ileum [6]. The cause behind the jejunal diverticular disease is unclear, however, it is thought that intestinal dysmotility plays a role. Because of this, congenital diseases, like systemic sclerosis and other visceral neuropathies, have an increased incidence of diverticular disease located in the jejunum [7].

Most patients with jejunal diverticula are asymptomatic, with incidental findings on CT. However, when patients are symptomatic, they usually present with chronic abdominal pain, early satiety, diarrhea, and bloating. The symptoms associated with jejunal diverticula are due to bacterial overgrowth and subsequent malabsorption [8]. Complications can be severe and life-threatening, ranging from small bowel obstruction secondary to diverticula, gastrointestinal bleeding in the form of hematochezia, and recurrent pancreatitis. Another complication is acute diverticulitis, which can lead to perforation. To diagnose small bowel diverticula, a CT scan is utilized, which will show globular outpouchings [9]. Diagnosis is confirmed by laparoscopy or an exploratory laparotomy procedure [10].

Treatment varies with respect to the severity of symptoms. Asymptomatic patients do not require treatment. If a patient presents with a small bowel obstruction secondary to impaction of the diverticula, the stone can be extracted or crushed with the treatment of a small bowel obstruction subsequently implemented, including nasogastric tube placement and surgical decompression if necessary. Furthermore, if a patient presents with diverticular bleeding, resuscitation is started, and bleeding is controlled [11].

This 80-year-old patient presented with chronic abdominal pain and was found to have perforated jejunal diverticulitis. Due to the low incidence of perforated small bowel diverticulitis, the guidelines for treatment follow that of colonic diverticulitis. When there’s evidence of perforation, surgery is the first line of treatment. This usually occurs in the setting of a Hartmann‘s procedure, in which the perforated portion of the bowel is removed, and a diverting colostomy is placed, with the intention of reversing the colostomy in the future. The secondary option is a one-stage surgery in which there is resection and a primary anastomosis placed. There is conflicting data on which surgical technique is better. For example, a systematic review done in 2006 showed decreased mortality with the one-stage surgery as opposed to Hartmann’s procedure [12]. All of this data is based upon colonic diverticula, making appropriate treatment modalities for small bowel manifestations uncertain.

Upon literature review, the patient's symptoms will direct the course of treatment. There have been cases that showed nonperforated localized peritonitis that was treated with broad-spectrum antibiotics, bowel rest, and percutaneous image-guided aspiration for localized intraperitoneal collection [10,13,14]. However, with complicated and perforated jejunal diverticulitis, immediate surgical intervention with segmental bowel resection and anastomosis is necessary [10].

When presented with this case, it is initially easy to question the treatment provided to this patient, especially given the outcome. Would this patient have fared better if treated with Hartmann’s procedure and left with a diverting ostomy as opposed to a primary anastomosis? Especially knowing our patient with his advanced age and significant past medical history that increased his risk of an anastomotic leak, hindsight may force us to question the choices made in the operating room. However, there are such limited studies done with small bowel diverticula, especially when concentrated in the jejunum, that it is difficult to compare appropriate treatment modalities. On top of this, going forward with an ostomy poses certain risks that are avoided with a primary anastomosis. When all of these factors are taken into consideration, primary anastomosis seems to be the best option at present.

## Conclusions

Small bowel diverticular disease is a rare manifestation of diverticular disease, especially when it is seen in the jejunum, as was seen in this patient. Because of the low incidence rate of this disease, guidelines are limited and not fully studied, making the assessment of this case difficult and leaving many questions unanswered. While this patient was treated with the best practices to date, the question remains if these are, indeed, the best practices. Further studies are needed to continue the discussion on small bowel diverticula and the practices and management strategies that will best serve patients.
